# Prévalence de l’antigène de surface du virus de l’hépatite B et facteurs associés chez des militaires sénégalais envoyés en mission au Darfour

**DOI:** 10.11604/pamj.2017.26.154.11594

**Published:** 2017-03-15

**Authors:** Moustapha Diop, Assane Diouf, Said Malaobé Seck, Gora Lo, Daye Ka, Aminata Massaly, Alassane Dieye, Ndeye Maguette Fall, Viviane Marie Pierre Cisse-Diallo, Khardiata Diallo-Mbaye, Ndèye Aissatou Lakhe, Louise Fortes-Déguénonvo, Cheikh Tidiane Ndour, Maserigne Soumaré, Moussa Seydi

**Affiliations:** 1 Service des Maladies Infectieuses et Tropicales, Chnu de Fann, Dakar, Sénégal; 2 Ecole de Santé Publique de l’Université de Montréal (ESPUM), Québec, Canada; 3 Service d’Ophtalmologie, Hôpital Principal, Dakar, Sénégal; 4 Centre Médical Inter Armée Sud, Dakar, Sénégal

**Keywords:** Hépatite B, militaires, Sénégal, Hepatitis B, military personnel, Senegal

## Abstract

**Introduction:**

Au Sénégal, 85% de la population adulte ont été en contact avec le virus de l'hépatite B et environ 11% sont porteurs chroniques de l'antigène de surface de ce virus (AgHBs). Cette infection est peu documentée dans l'armée sénégalaise. L'objectif de cette étude était d'évaluer la prévalence de l'AgHBs chez des militaires sénégalais envoyés en mission au Darfour (Soudan) et d'identifier les facteurs associés.

**Méthodes:**

Nous avons mené une étude transversale du 1^er^ juillet 2014 au 31 juillet 2014 chez des militaires sénégalais en mission au Darfour. La recherche de l'AgHBs a été effectuée dans le sérum des participants par la méthode immunochromatographique. La recherche de facteurs associés a été réalisée à l'aide d'une régression logistique multivariée.

**Résultats:**

Notre étude a porté sur 169 militaires de sexe masculin. L'âge moyen était de 36,6 ans ± 9,5. Des antécédents d'hépatopathie chronique au niveau familial, d'exposition sanguine et d'exposition sexuelle ont été retrouvés respectivement chez 12,4% ; 24,9% et 45,6% de la population d'étude. L'AgHBs a été retrouvé chez 24 participants [14,2% (IC95% = 8,9-19,5)]. Après ajustement sur les facteurs de confusion potentiels, l'âge (OR=0,9 IC95% =0,9-1,0), un niveau d'étude universitaire (OR= 9,5 IC95% =1,3-67,1) et l'exposition sexuelle (OR=3,3; IC95% =1,0-10,3) étaient apparus associés de façon indépendante à l'hépatite B.

**Conclusion:**

Notre étude retrouve une prévalence élevée de l'AgHBs et souligne la nécessité d'une évaluation plus poussée de l'hépatite B chez cette population.

## Abstract (en)

**Introduction:**

In Senegal, 85% of the adult population have been exposed to the hepatitis B virus and about 11% of them are chronic surface antigen (HBsAg) carriers. This infection is poorly documented among Senegalese Armed Forces. The aim of this study was to assess the prevalence of HBsAg in Senegalese military personnel on mission to Darfur (Sudan) and to identify its associated factors.

**Methods:**

We conducted a cross-sectional study among Senegalese military personnel stationed in Darfur from 1 July 2014 to 31 July 2014. HBsAg test was performed on serum of participants using immunochromatographic method. The search for associated factors was carried out using multivariate logistic regression.

**Results:**

Our study included 169 male military personnel. The average age was 36.6 ± 9.5 years. A history of familial chronic liver disease, blood exposure and sexual exposure were found in 12.4%, 24.9% and 45.6% of the study population respectively. HBsAg was found in 24 participants [14.2% (CI 95% = 8.9-19.5)]. After adjusting for potential confounding factors, age (OR = 0.9 CI 95% = 0.9-1.0), university level (OR = 9.5 CI 95% = 1.3 - 67 , 1>) and sexual exposure (OR = 3.3 <; CI 95% = 1.0 - 10.3) were independently associated with hepatitis B.

**Conclusion:**

Our study shows high prevalence of HBsAg and underlines the need for further evaluation of hepatitis B in this population.

## Introduction

L'hépatite B constitue un problème mondial de santé publique. Le nombre de personnes souffrant d´une infection chronique par le virus de l'hépatite B (VHB) est estimé à 240 millions parmi lesquelles 650 000 meurent chaque année des complications de cette infection [1]. La prévalence du portage de l'antigène de surface du virus de l'hépatite B (AgHBs) est très variable selon la zone géographique et l'Afrique subsaharienne fait partie des zones ayant la prévalence la plus élevée. La proportion de la population adulte atteinte d'une hépatite B chronique y est supérieure à 5% [1, 2]. Au Sénégal, 85 % de la population générale ont au moins un marqueur du VHB [3, 4] et la prévalence l'AgHBs évaluée dans plusieurs groupes de populations d'intérêt variait entre 7,35% et 14% [5–10]. Parmi ces groupes, une enquête nationale a récemment rapporté une prévalence de 10,8% chez les militaires [11]. Dans cette population, une certaine catégorie est sélectionnée pour des missions à l'étranger. Elle présente quelques particularités épidémiologiques : elle est en meilleure santé apparente, sexuellement active et séjourne à l'étranger pendant plusieurs mois sans accompagnement conjugal. C'est dans ce contexte que nous avons mené cette étude. Elle avait pour objectif d'évaluer prévalence de l'AgHBs chez des militaires sénégalais envoyés en mission de maintien de la paix au Darfour / Soudan et de déterminer les facteurs associés au portage de l'AgHBs.

## Méthodes

**Figure 1:**
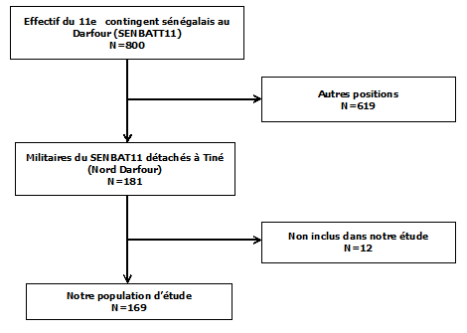
Diagramme de flux du contingent de militaires sénégalais envoyés au Darfour en 2014

Nous avons réalisé une étude transversale descriptive et analytique allant du 1^er^ juillet 2014 au 31 juillet 2014. Notre travail a porté sur une population de militaires issue du 11e contingent sénégalais envoyée en mission de maintien de la paix au Darfour (Soudan) selon le diagramme de flux suivant (Figure 1). Nous avons inclus dans notre étude les militaires qui étaient détachés à TINE (Nord Darfour) et chez qui la recherche d'AgHBs dans le sang a été réalisée durant leur visite médicale d'aptitude. Cette visite médicale a été réalisée dans les 4 mois qui précédaient la mission. Nous n'avons pas inclus dans notre travail les militaires chez qui l'entretien directif individuel n'a pas été réalisé. Nos données ont été recueillies à partir de l'exploitation du dossier médical de chaque militaire et grâce à des entretiens directifs individuels. Il s'agissait de données socio-démographiques, des antécédents familiaux d'hépatopathies chroniques (hépatite B, cirrhose du foi, cancer du foie), de la notion de voyage à l'étranger, d'une vaccination antérieure contre l'hépatite B, de la notion d'exposition sanguine, de la notion d'exposition sexuelle, de la notion de consommation de drogues, des données cliniques, des données paracliniques (AgHBs, ASAT, ALAT entre autres) et du traitement en cours La recherche de l'AgHBs a été effectuée dans le sérum des participants par la méthode immunochromatographique sur plaque. La réaction antigène-anticorps, se traduisant par l'apparition d'un trait coloré en rose, signait la positivité de l'antigénémie HBs. Les données ont été saisies sur une base Epidata version 3.1 et analysées grâce à la version 12 du logiciels Stata. Les comparaisons de proportions ont été réalisées par le test du chi-carré ou le test exact de Fisher si nécessaire. Les moyennes ont été comparées à l'aide du test T de Student, de l'ANOVA ou du test de Krusskal Wallis. La recherche de facteurs associés a été réalisée à l'aide d'un modèle de régression logistique. Les différences ont été considérées comme significatives lorsque la valeur de p correspondante était inférieure à 0,05.

## Résultats

**Figure 2:**
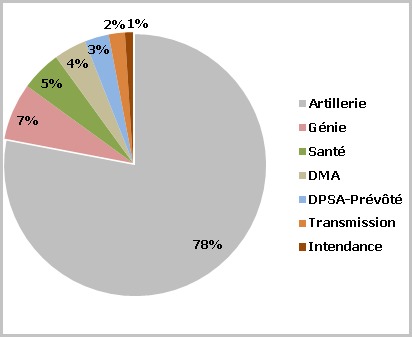
Répartition de la population d’étude selon le corps d’appartenance

**Répartition de la population d'étude selon le corps d'appartenance:** Plusieurs corps étaient représentés dans notre population mais la majeure partie était constituée de militaires du bataillon de l'artillerie (Figure 2).

**Caractéristiques de la population d'étude:** Les caractéristiques de notre population d'étude sont résumées dans le Tableau 1. Notre population d'étude était exclusivement masculine avec des âges extrêmes de 23 ans et 54 ans. Parmi les 119 militaires qui avaient voyagé à l'étranger, 116 avaient séjourné en Afrique occidentale ou en Afrique centrale et 6 en Afrique du Nord. Les voyages dans les autres régions du monde étaient rares. Le nombre de voyages par participant variait de 1 à 4 et la durée totale des séjours à l'étranger variait de 1 à 38 mois. Pour plus de 90% des participants, l'objet du voyage incluait une mission militaire. Un effectif de 51 militaires ne pouvait pas fournir l'information sur une vaccination antérieure contre le VHB. Pour les 20 militaires vaccinés contre l'hépatite B, le nombre de doses reçues a été précisé chez 18 individus et variait de 1 à 4. La notion de consommation d'alcool a été rapportée par 34 participants (20,12%). Parmi eux, le nombre moyen d'années de consommation d'alcool était précisé chez 22 personnes et était de 15,8 ± 11,9. Le même effectif avait précisé le nombre moyen de verres par semaine qui était de 5,7 ± 9,0. Vingt et huit militaires étaient des fumeurs actifs et leur nombre moyen de paquet année était de 4,8. Les anciens fumeurs représentaient 30,18% de la population avec un nombre moyen de paquet année à 3,3.

**Tableau 1:** Caractéristiques de la population d’étude

Variables	Moyenne (an) / effectif	Ecart type/ %
**Age**	36,6	9,5
**Durée de service militaire**	14,3	9,4
**Situation matrimoniale**		
Mariés	114	67,5
Célibataires	55	32,5
**Niveau d’étude**		
Primaire	33	19,5
Secondaire	126	74,6
Universitaire	10	59,2
**Grade**		
Officiers	3	1,8
Sous-officiers	61	36,1
Militaires du rang	105	62,1
**Voyage antérieur**		
Oui	119	70,4
Non	50	29,6
**Antécédent pathologie hépatique (hépatite B, cirrhose, cancer)**		
Oui	21	12,4
Non	145	85,8
Ne sait pas	3	1,8
**Antécédent exposition sanguin**		
Oui	42	24,9
Non	127	75,1
**Antécédent exposition sexuel**		
Oui	77	45,6
Non	92	54,4
**Vaccination contre VHB**		
Oui	20	11,8
Non	98	57
Ne sait pas	51	30,2
**Alcool**		
Oui	34	20,1
Non	135	79,9
**Tabac**		
Oui, toujours	28	16,6
Oui, mais arrêt	51	30,2
Jamais	90	45,9

**Prévalence de l'antigène HBs et facteurs associés:** Dans notre population d'étude, 24 militaires avaient une antigénémie HBs positive soit une prévalence à 14,2% (IC95% ; 8,9-19,5). Parmi les 20 militaires qui étaient vaccinés contre l'hépatite B, aucun ne présentait une antigénémie HBs positive. Cette prévalence était variable selon les catégories d'âge. [23-26 ans] : 25%, 27-37 ans] : 8,5%, [38-43 ans] : 15,8%, [44-54 ans] :10,4% (p=0,14). Elle variait aussi selon le niveau d'étude avec une différence statistiquement significative (primaire : 6%, secondaire : 14,3% et universitaire : 40% avec un p à 0,027). Pour les autres paramètres étudiés la différence de la prévalence n'était pas statistiquement significative. Après ajustement sur les facteurs de confusion potentiels, l'âge jeune {[27-37ans]:OR=0,16 ; IC95%=0,04-0,68 ; p=0,012, [38-43ans]:OR=0,22 ; IC95%=5,11-96,01 ; p=0,044 ≥ 44ans: OR=0,11; IC95%=2,22-50,49 ; p=0,005}, le niveau d'étude (OR= 12,11 IC95% ;1,53-95,96 ; p=0,02) et l'exposition sexuelle (OR=3,0 ; IC95% ; 1,0-10,3;p=0,04) étaient apparus associés de façon indépendante au portage de l'AgHBs (Tableau 2).

**Tableau 2:** Régression logistique univariée et multivariée évaluant les facteurs associés au portage de l’AgHBs chez les militaires sénégalais envoyés en mission au Darfour en 2014

	Analyse univariée		Analyse multivariée	
Caractéristiques de la population	Odds ratio (IC95%)	p-value	Odds ratio (IC95%)	p-value
**Tranche d’âge (ans)**				
<27 ans	1		1	-
27 – 37	0,28(0,08 - 1,00)	0,049	0,16 (0,04 – 0,68)	0,013
38 – 43	0,56(0,18 –1,782)	0,328	0,22(5,11 – 96,01)	0,044
≤ 44	0,35(0,11 - 1,15)	0,084	0,11(2,22 – 50,49)	0,005
**Durée de service militaire**	0,96 (0.92 -1.01)	0,147		
**IMC (kg/m^2^)**	1,02 (0,87 – 1,19)	0,840	-	-
**Grade**			-	-
Officiers	1		-	-
Sous-officiers	0,22 (0,02 – 2,78)	0,241	-	-
Militaires de rang	0,39 (0,03 – 4,50)	0,448	-	-
**Situation matrimoniale**			-	-
Marié	1		-	-
Célibataire	1,88 (0,78 – 4,52)	0,158	-	-
***Niveau d’étude***				
Primaire	1			
Secondaire	2,58 (0.57 – 11,75)	0,219	2,06 (0,42 – 10,02)	0,370
Universitaire	10,33 (1,53 – 69,73)	0,017	12,11(1,53 – 95,96)	0,018
**Voyage antérieur** ^&^	0,43 (0,18 – 1,05)	0,064		
**Antécédents familiaux** ^&^	2,07 (0,68 – 6,31)	0,200	2,93 (0,81 -10,61)	
**Antécédents d’exposition sanguine** ^&^	1,29 (0,50 – 3,38)	0,598	-	-
***Antécédents d’exposition sexuelle*** *^&^*	1,49 (0,63 – 3,56)	0,363	3,26 (1,03 – 10,34)	0,044
**Consommationd’alcool** ^&^	0,77 (0,24 – 2,41)	0,650	-	-
**Tabagisme** ^&^	0,96 (0,40 – 2,28)	0,923	-	-
**ALAT > 50 UI/ml** ^&^	2,40 (0,57 – 10,17)	0,233	-	-
**ASAT > 50 UI/ml** ^&^	5,35 (0,32 – 89,10)	0,243	-	-

&=présence/absence,β=par augmentation d’une unité

## Discussion

Notre étude retrouve une séroprévalence de l'AgHBs de 14,2% dans une population de 169 militaires sénégalais envoyés en opération externe au Darfour. Cette prévalence, supérieure à 8%, place notre population d'étude dans le groupe de forte prévalence selon la classification de l'OMS [1]. Les facteurs associés de façon indépendante au portage de l'AgHBS étaient l'âge, les antécédents d'exposition sexuelle et le niveau d'étude Notre étude s'est intéressée à une population exclusivement masculine et tous les facteurs de confusion potentiels connus et mesurables à notre niveau ont été pris en compte. Comme toute étude transversale, elle présente des limites parmi lesquelles l'impossibilité d'établir des relations causales entre les expositions et l'événement. Par exemple, aucun militaire précédemment vacciné n'avait une antigénémie HBs positive mais le devis de l'étude ne permet pas de préciser la séquence temporelle entre la vaccination et l'antigénémie HBs. De même, comme dans la plupart des études, nous n'avons fait que le dosage de l'antigène de surface du virus de l'hépatite B (AgHBs). Nous n'avons pas dosé les autres marqueurs du virus de l'hépatite B tels que l'Ag Hbe (preuve d'une réplication virale), les anticorps anti-HBc et l'ADN viral. Le dosage des anticorps anti-HBc de type IgM et IgG nous aurait permis de faire la distinction entre les infectés récents et les porteurs chroniques. La recherche de l'ADN virale de l'hépatite B pourrait nous permettre de trouver une prévalence plus élevée en mettant en évidence les cas d'hépatite B occulte [12].

La prévalence retrouvée dans cette population d'étude est supérieure à celle notée dans un échantillon aléatoire de militaires sénégalais qui était de 10,8% [11]. Elle est également supérieure à celle retrouvée dans la plupart des populations d'intérêt au Sénégal : 7,35% ; 7,9% ; 8,8% ; 11,6% ; 13,9% et 14 ,0% respectivement chez les donneurs de sang, les consommateurs de drogues injectables (CDI), les personnes vivant avec le VIH (PVVIH), les femmes enceintes, les homosexuels et les prisonniers [5–10]. Le caractère exclusivement masculin de notre population d'étude peut contribuer à expliquer cette différence. En effet la prédominance de l'hépatite B chez les sujets de sexe masculin est constamment décrite dans la littérature. Cette prédominance masculine a été notée par Ott J et al lors d'une revue épidémiologique à échelle mondiale sur l'hépatite B [13]. Elle a été aussi retrouvée par LO G et Ndiaye A respectivement chez les PvVIH et la population militaire au Sénégal [8, 11]. Cette prédominance masculine serait liée en partie à un facteur génétique protégeant la femme contre l'infection au virus de l'hépatite B. La prévalence retrouvée dans notre étude est légèrement inférieure à celle retrouvée chez le même type de population en Côte d'ivoire qui appartient aussi à la zone de forte prévalence selon l'OMS [1]. Ainsi dans une population masculine de gendarmes ivoiriens, une prévalence de 15,6 % du portage de l'Antigène HBs a été retrouvée par Kra O et al [14].

Dans notre étude, L'exposition sexuelle était indépendamment associée au portage de l'antigène de surface du virus de l'hépatite B [OR = 3,26, IC95%(1,0-10,3);p=0,04]. Ce résultat est superposable à celui de Chacaltana A et al aux États Unies en 2008(OR: 6,3; IC 95%:1,7-23.4 ; p=0.006) et Birku T et al en Ethiopie en 2015 (OR 4,3; 95 % IC 1.1-16,4 ; p = 0,03) chez des populations militaires [15, 16]. De même, Anaedobe CG et al avaient trouvé le multi partenariat sexuel comme un facteur associé à l'infection par le virus de l'hépatite B chez des femmes enceintes au Nigéria (OR=3,987 ; p-value=0,026) [17]. Cette association s'explique par le fait que la voie de transmission de l'hépatite B chez l'adulte soit essentiellement sexuelle. L'âge jeune était aussi identifié comme facteur associé au portage de l'AgHBs dans notre étude {[27-37ans]: OR=0,16 ; IC95%=0,04-0,68 ; p=0,012, [38 ≥ 43ans]:OR=0,22 ; IC95%=5,11-96,01 ; p=0,044 ≥ 44ans: OR=0,11; IC95%=2,22-50,49 ; p=0,005}. Un résultat similaire, aussi trouvé par Ndiaye A et al au Sénégal chez la population de militaires sénégalais [11], concorde avec les ceux de Lemoine M et al en Afrique [18]. Elle peut être expliquée par plusieurs raisons : - la possibilité d'une guérison spontanée de l'hépatite B entrainant ainsi une disparition de l'antigène HBs - la possibilité d'un passage à la chronicité puis la survenue de décès - le fait que le sujet jeune soit plus exposé à des facteurs de risque de l'hépatiteB. Par ailleurs l'association du niveau d'étude élevé au portage de l'AgHBs retrouvée dans notre étude (OR= 12,11 IC95% ; 1,53-95,96 ; p=0,02) n'est pas reconnue dans la littérature. Metaferia Y et al avaient d'ailleurs noté un résultat contraire chez des femmes enceintes en Ethiopie en 2015 (OR=3,68;95%IC ; 1,268-10,678; p=0,017) [19]. D'autres facteurs connus comme associés à l'hépatite B tels que les antécédents familiaux d'hépatopathie chronique et l'exposition sanguine n'ont pas été notés dans notre étude. Cependant l'OR pour les antécédents familiaux d'hépatopathie chronique était de 3,16 (0,87-11,46) avec 21 cas dans notre population d'étude. Ce qui explique le déficit de puissance pour mettre en évidence une association statistiquement significative.

## Conclusion

Dans cette population de militaires, la prévalence élevée du portage de l'Antigène HBs semble indiquer un problème d'une ampleur plus importante que dans la plupart des autres groupes de population au Sénégal. Il est donc nécessaire d'explorer plus en profondeur cette problématique pour au moins déterminer : la part des hépatites actives, l'effectif ayant besoin d'un traitement et l'effectif ayant besoin d'une vaccination contre le VHB. Ce qui participera à un meilleur contrôle de l'infection par le VHB dans cette population.

### Etat des connaissances actuelles sur le sujet

- L'hépatite B est un problème mondial de santé publique et l'Afrique de l'ouest en est une zone de forte prévalence. Les facteurs associés à cette infection sont bien connus dans la littérature ;
- Cette maladie n'est pas bien documentée dans la population militaire africaine.

### Contribution de notre étude à la connaissance

- Notre étude montre une prévalence élevée de l'antigène de surface du virus de l'hépatite B et identifie quelques facteurs associés
- Notre étude souligne la nécessité d'une investigation plus poussée sur cette infection en faisant un dosage exhaustif des marqueurs viraux chez les militaires. Elle souligne aussi l'intérêt d'une meilleure sensibilisation sur cette maladie accès surtout sur les facteurs associés.
